# Unmapped exome reads implicate a role for *Anelloviridae* in childhood HIV-1 long-term non-progression

**DOI:** 10.1038/s41525-021-00185-w

**Published:** 2021-03-19

**Authors:** Savannah Mwesigwa, Lesedi Williams, Gaone Retshabile, Eric Katagirya, Gerald Mboowa, Busisiwe Mlotshwa, Samuel Kyobe, David P. Kateete, Eddie Mujjwiga Wampande, Misaki Wayengera, Sununguko Wata Mpoloka, Angella N. Mirembe, Ishmael Kasvosve, Koketso Morapedi, Grace P. Kisitu, Adeodata R. Kekitiinwa, Gabriel Anabwani, Moses L. Joloba, Enock Matovu, Julius Mulindwa, Harry Noyes, Gerrit Botha, Collaborative African Genomics Network (CAfGEN), Masego Tsimako-Johnstone, Fred. A. Katabazi, Edgar Kigozi, Keofentse Mathuba, TrypanoGEN Research Group, Chester W. Brown, Graeme Mardon, Mogomotsi Matshaba, Neil A. Hanchard

**Affiliations:** 1 College of Health Sciences, Makerere University, Kampala, Uganda; 2 University of Botswana, Gaborone, Botswana; 3 Baylor College of Medicine Children’s Foundation Uganda (Baylor Uganda), Kampala, Uganda; 4 Botswana-Baylor Children’s Clinical Centre of Excellence, Gaborone, Botswana; 5 College of Veterinary Medicine, Animal Resources and Biosecurity, Makerere University, Kampala, Uganda; 6 Institute of Integrative Biology, University of Liverpool, Liverpool, UK; 7 Institute of Infectious Disease and Molecular Medicine, University of Cape Town, Cape Town, South Africa; 8 University of Tennessee Health Science Center, Le Bonheur Children’s Hospital, Memphis, TN USA; 9 Department of Molecular and Human Genetics, Baylor College of Medicine, Houston, TX USA; 10 Present Address: National Human Genome Research Institute, National Institutes of Health, Bethesda, MD USA

**Keywords:** Viral genetics, Risk factors

## Abstract

Human immunodeficiency virus (HIV) infection remains a significant public health burden globally. The role of viral co-infection in the rate of progression of HIV infection has been suggested but not empirically tested, particularly among children. We extracted and classified 42 viral species from whole-exome sequencing (WES) data of 813 HIV-infected children in Botswana and Uganda categorised as either long-term non-progressors (LTNPs) or rapid progressors (RPs). The Ugandan participants had a higher viral community diversity index compared to Batswana (*p* = 4.6 × 10^−13^), and viral sequences were more frequently detected among LTNPs than RPs (24% vs 16%; *p* = 0.008; OR, 1.9; 95% CI, 1.6–2.3), with *Anelloviridae* showing strong association with LTNP status (*p* = 3 × 10^−4^; *q* = 0.004, OR, 3.99; 95% CI, 1.74–10.25). This trend was still evident when stratified by country, sex, and sequencing platform, and after a logistic regression analysis adjusting for age, sex, country, and the sequencing platform (*p* = 0.02; *q* = 0.03; OR, 7.3; 95% CI, 1.6–40.5). Torque teno virus (TTV), which made up 95% of the *Anelloviridae* reads, has been associated with reduced immune activation. We identify an association between viral co-infection and prolonged AIDs-free survival status that may have utility as a biomarker of LTNP and could provide mechanistic insights to HIV progression in children, demonstrating the added value of interrogating off-target WES reads in cohort studies.

## Introduction

The rates of new human immunodeficiency virus (HIV) infection in Eastern and Southern Africa increased by 50% between 2015 and 2019, despite a modest overall decline in new infections globally^1,2^. As such, this region still holds a disproportionate burden of new HIV infections in children, representing 53% of the global burden^2^.

Two extreme clinical phenotypes characterise HIV-1 progression in children: (1) long-term non-progressors (LTNPs), who do not progress to AIDS for more than 10 years without antiretroviral therapy (ART) and (2) rapid progressors (RPs), who typically advance to AIDS less than 3 years after initial infection^3^. Studies examining the molecular mechanisms underlying HIV-1 disease progression in adults suggest a complex interplay between host genetics^4–8^, alongside immunological, virological^9^, and nutritional factors^10–12^. Insights gained from understanding these contributing factors have heralded the development of new HIV drugs^13^. Most of these studies, however, have focused on Caucasian populations, even though the underrepresented African populations that bear the greatest burden of this scourge display some of the most diverse genetics in addition to unique environmental exposures^14^, which could yield new insights to HIV-1 pathogenesis. In addition, few studies have shed light upon the molecular regulators of HIV-1 disease progression in children, who are both immunologically and developmentally distinct from their adult counterparts. For example, CD4^+^ and CD8^+^ T-cell activation in HIV-1 infected children correlates significantly with CD4 T-cell percentage or absolute count, rather than viral load, which correlates strongly in adults^15^. Another recent study demonstrated that paediatric non-progressors exhibited low immune activation despite high viral loads^16^, in contrast to adult elite controllers in whom the converse is true^15^.

Chronic infection by RNA and DNA viruses often results in the production of antiviral cytokines, including interferons, which can lead to tightly regulated inflammatory responses in the host^17^. This modified state of host immunity or immune modulation comprises both increased (immune potentiation) or decreased (immune suppression) immunity^18^, which, in turn, may alter susceptibility to inflammatory diseases^19,20^. Such co-infection may also offer unexpected benefits to the host, e.g. prior dengue virus infection may reduce the risk of symptomatic Zika in paediatric populations^21^, and there is an established association between human T-cell lymphotropic virus type II co-infection and delayed AIDS progression^22,23^. To date, however, there have been no robust assessments of viral co-infection in disease progression among HIV positive children.

As a targeted resequencing approach, whole-exome sequencing (WES) allows for the enrichment of the exonic sequences from genomic DNA (gDNA). An offshoot of such targeted resequencing approaches are “off-target” reads, i.e. sequencing reads that have been captured but do not map to the targeted regions; over 50% of reads may be “off-target” (including reads that map to introns and intergenic regions) and may include reads representative of free viral DNA or viral DNA that is integrated into the host genome (proviral DNA)^24,25^. A recent analysis of whole-genome sequencing (WGS) from 8000 human genomes found that ~0.01% of reads mapped to viral reads. Despite the relatively small fraction, the cumulatively large absolute amount of data was sufficient for a broad characterisation of the blood virome^26^. Other studies have also utilised off-target viral reads from tumour sequencing to investigate the association between virus species and cancer types^27,28^. Here, we extract viral reads from often-ignored unmapped data to explore the role of viral co-infection in paediatric HIV-1 disease progression in two African cohorts of LTNPs and RPs recruited through a unique electronic health record resource in Uganda and Botswana. We provide evidence for a robust recapitulation of the virome from unmapped WES reads and demonstrate enrichment of co-infecting viral species based on geographic location and HIV-1 disease progression.

## Results

### Overall characteristics of unmapped reads

We analysed unmapped reads from WES data of 813 samples collected from the CAfGEN cohort (Table 1).

**Table 1:** Summary of the cohort.

Variable	Overall, *n* = 813	LTNP, *n* = 391^a^	RP, *n* = 422^a^	*p* value^b^
Country				<0.001
Botswana	450 (55%)	189 (48%)	261 (62%)	
Uganda	363 (45%)	202 (52%)	161 (38%)	
Sex				<0.001
Female	421 (52%)	228 (58%)	193 (46%)	
Male	392 (48%)	163 (42%)	229 (54%)	
Time to progression (range) in months	36 (16, 155)	156 (138, 177)	17 (10, 25)	<0.001
Age at enrolment (range) in months	152 (111, 198)	200 (164, 232)	116 (78, 145)	<0.001
Duration of HAART at enrollment (range) in months	63 (17, 106)	19 (2, 63)	98 (61, 125)	<0.001

Demographic variables of the sampled populations from Uganda and Botswana.

^a^Statistics presented: *n* (%); median (IQR).

^b^Statistical tests performed: Fisher’s exact test; Wilcoxon rank-sum test.

**Fig. 1:**
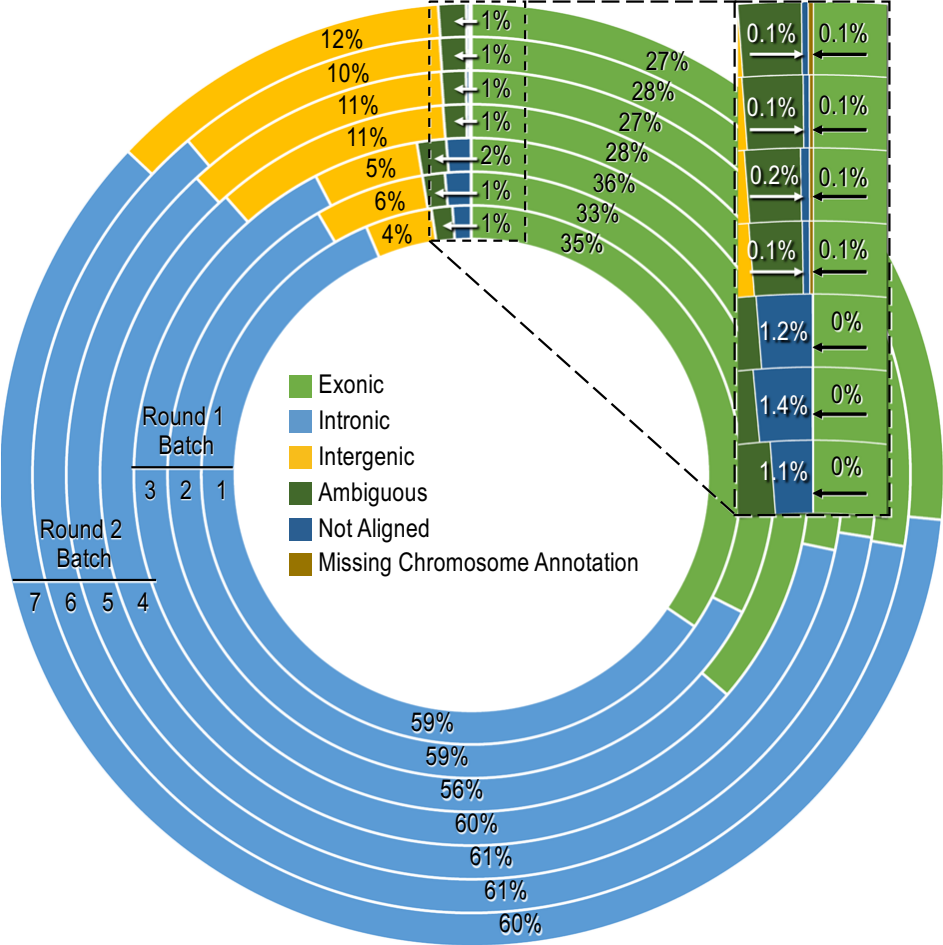
Summary percentage distribution of raw reads after aligning to the reference genome. WES was carried out in seven batches, and after aligning raw reads to the human reference genome (hg19), BAM files were analysed for reads mapping inside the genomic regions utilising annotations from Ensembl (www.ensembl.org). Although 30% of the 5.7 × 10^10^ raw reads mapped exclusively to the exons, 66% of the reads that mapped to introns or intergenic reads also overlapped with exons (not shown). Round 1 batches (inner three rings), sequenced on the Illumina HiSeq 2500 platform, had a higher percentage of unmapped reads (1.2%) compared to the round 2 batches (outer four rings), sequenced on the Illumina NovaSeq 6000 platform (0.2%). Overall, 3.0 × 10^8^ reads (~0.6% of the total reads) did not map to the human reference genome (inset).

Consistent with earlier reports^29–31^, ~75% of the reads were on-target, with 30% mapping exclusively to the targeted exons, and 68% generally mapping to intronic/intergenic regions (66% of these intronic/intergenic reads also overlapped the exons). Overall, 1% of the reads had ambiguous alignments or missing annotations, and 0.6% did not map to the UCSC hg19 human genome sequence; these unmapped reads potentially contained reads from viral nucleic acid sequences (Fig. 1).

**Fig. 2:**
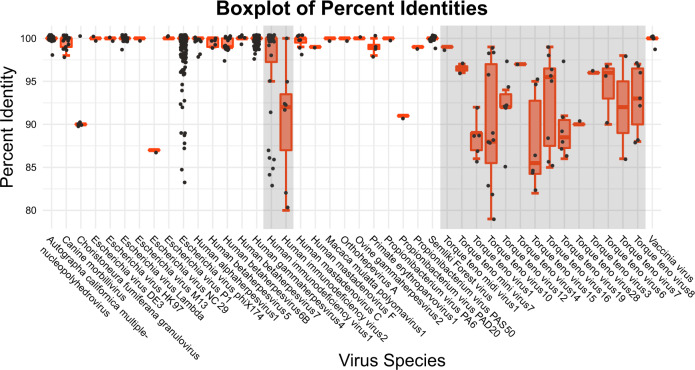
Boxplots of viral-contig percent identities compared to the reference virus dataset. All viruses had a median identity of >85%. HIV and Torque-Teno Virus (TTV) show higher variability in the identity of sequences (shaded grey), suggesting higher diversity of these species. The boxplot represents the median values (centre lines), first and third quartiles (bounds of boxes), and the whiskers indicate 1.5 times the interquartile range.

Across all participants, detected viruses could be mapped to 42 different species (Supplementary Data 1). The median identity across all detected viruses was over 85% (Fig. 2), which is above the 70% threshold suggested to target reads from viruses of the same taxonomical subfamily^32^. The mean BLASTN *E*-value was 1 × 10^−11^, and the mean bit-score was 4000 for viral contigs (excluding phiX174, an intra-experimental control).

**Fig. 3:**
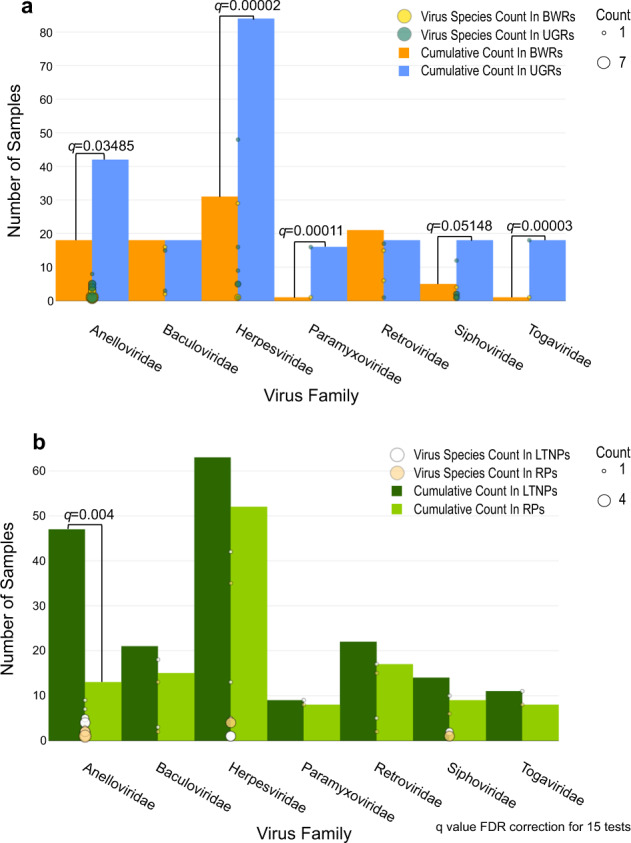
A comparison of the distribution of viral reads between Uganda (UGR, *n* = 117) and Botswana (BWR, *n* = 64) cohorts and between LTNPs (*n* = 104) and RPs (*n* = 77). **a** Compared to Botswana, more Ugandan samples had reads from *Anelloviridae*, *Herpesviridae*, *Paramyxoviridae*, *Siphoviridae*, and *Togaviridae*. **b** LTNPs had a higher frequency of *Anelloviridae*, with this trend being consistent across all the virus families, though not statistically significant. Bubble size is proportional to the number of virus subtypes. Only viral families that were found in more than 1% of the samples were considered. Fisher’s exact test was used to compute *p* values and adjusted *p* values (*q*-values) were calculated using FDR method.

Besides phiX DNA, the most common virus was human herpesvirus (types 1, 4, 5, 6B, 7), detected in 10% of the samples (Supplementary Data 1). HIV (*Retroviridae*) was the second most common virus detected. Although all participants were confirmed as HIV positive, we were only able to detect HIV in 4.6% (38/812) of samples, suggesting that most HIV proviral DNA reads are either outside of the coding region or lost during the exome enrichment step. Nonetheless, the distribution of HIV proviral reads was not significantly different between LTNPs and RPs (*p* = 0.408; odds ratio (OR), 1.4; 95% confidence interval (CI), 0.7–2.8 by Fisher’s exact test) or between countries (Uganda vs Botswana; *p* = 0.741; OR, 1.1; 95% CI, 0.6–2.3 by Fisher’s exact test) (Fig. 3).

Because we used a DNA sequencing protocol, the presence of RNA viruses such as Semliki forest virus (*Togaviridae*) and HIV was surprising (Supplementary Data 1). The incidental integration of non-retroviral RNA viruses has been suggested, however, the mechanism for *Togaviridae* remains controversial^33^, as such, its presence may be explained by background contamination from reagents^34^. However, the presence of HIV, a retrovirus, may point to proviruses and possible viral integration events^26^. Identifying host-integration sites is of interest:, as such sites play a role in disease progression^35^; however, due to the low number of viral reads, we could not detect chimeric host-viral reads and, therefore, could not identify HIV integration sites into the host genome.

### Viral diversity varies with country of origin and time to progression

We observed a higher frequency of *Herpesviridae* and *Anelloviridae* among Ugandan samples than Batswana (i.e. individuals from Botswana), with the Uganda cohort showing a higher diversity index than the Botswana cohort for overall viral reads detected (*p* = 4.62 × 10^−13^, Supplementary Table 2).

We also found that more LTNP than RP participants had any virus family detected (Fig. 3b). To investigate this further, we first excluded viral families that were found in less than 1% of the samples and then compared the number of samples with any virus family detected; this confirmed our original observation as significantly more LTNP than RP samples had a detectable virus (Table 2) (*p* = 0.008 OR, 1.6; 95% CI, 1.1–2.3 by Fisher’s exact test). When we stratified this analysis by country, we observed the same trend; however, it was only statistically significant in Botswana (*p* = 0.011; OR, 2.07; 95% CI, 1.2–3.6; Uganda *p* = 0.726; OR, 1.1; 95% CI, 0.7–1.8 by Fisher’s exact test) (Table 2). We also identified a higher diversity of viruses among LTNPs (*p* = 0.008, Hutcheson *t*-test^36^, Supplementary Table 2).

**Table 2:** Number of samples with a virus (of any family) detected in LTNPs and RPs.

Dataset	LTNP (*n* = 391)	RP (*n* = 422)	*p* value^a^	OR (95% CI)
Uganda	59/202 (29%)	44/161 (27%)	0.73	1.10 (0.68–1.79)
Botswana	34/189 (18%)	25/261 (10%)	0.01	2.07 (1.19–3.61)
Combined	93/391 (24%)	69/422 (16%)	0.01	1.60 (1.13–2.26)

*p* values and odds ratios were computed to compare LTNPs and RPs for differences in the distribution of samples with a virus detected. Frequency comparisons are based on viral families found in more than 1% of the study samples.

^a^Statistical tests performed: Fisher’s exact test.

### *Anelloviridae* species are enriched among LTNPs

**Fig. 4:**
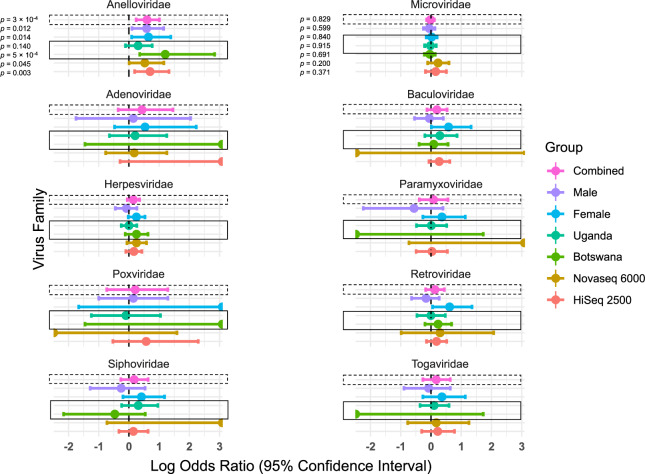
Forest plot of the effect size of the virus families on disease progression. Log odds ratios (95% CI) for the association of the virus families with LTNP status are plotted for the combined datasets (dashed-line box), when stratified by sex, stratified by country (solid-line box), and stratified by the sequencing platform used (NovaSeq 6000 or HiSeq 2500). The *Anelloviridae* showed the most significant association with LTNP status (top left). Microviridae (phiX) reads, used as a quality check for potential sequencing biases, show no association between LTNPs and RPs (top right). Only viral families found in more than one sample were considered; *p* values, odds ratios, and 95% CI were calculated using Fisher’s exact test.

*Anelloviridae* showed a strong enrichment among LTNPs compared to RPs in our dataset and included 12 different subtypes of torque teno virus (TTV), one subtype of torque teno mini virus (TTMV), and one subtype of Torque teno midi virus (TTMDV) in 38 individuals. These were significantly enriched among LTNPs (*p* = 3 × 10^−4^; *q* = 0.004, OR, 3.99; 95% CI, 1.74–10.25 by Fisher’s exact test) irrespective of the sequencing platform used (Figs 3b and 4) and were orthogonally validated using direct PCR (Supplementary Fig. 1) and Sanger sequencing where the samples clustered with genogroup 1 TTV (Supplementary Fig. 2).

We tested for an association of *Anelloviridae* detection with age, using age at sampling rather than age at diagnosis, and found that although individuals that had *Anelloviridae* detected were older than individuals that did not have *Anelloviridae* detected, this difference was non-significant, albeit marginally (median age, 14 years vs 12 years; Wilcoxon rank-sum test *p* = 0.05). Nevertheless, the association between LTNP status and *Anelloviridae* presence remained apparent even after a logistic regression analysis using a generalised linear model (GLM) with a binomial distribution, incorporating age at sample collection, sex, country of origin, and sequencing platform as covariates (*p* = 0.02; *q* = 0.03; OR, 7.3; 95% CI, 1.6–40.5). When we stratified our data according to the country of origin, we observed some evidence of replication between both country groups (*p* = 9 × 10^−4^; OR, 3.6; 95% CI, 1.6–9.3; by the Cochran–Mantel–Haenszel (CMH) chi-squared test), with substantial supporting evidence in Botswana (*p* = 5 × 10^−4^; OR, 16.0; 95% CI, 2.3–691.3) and the same directional trend in Uganda (i.e. more in the LTNPs than RPs), although the latter did not meet our threshold for statistical significance (*p* = 0.140; OR, 2.0; 95% CI, 0.8–5.9) (Fig. 4). In addition, the same trend was observed after stratifying by sex (*p* = 2 × 10^−4^; OR, 4.1; 95% CI, 1.8–10.6; by the CMH chi-squared test) in both females (*p* = 0.014; OR, 4.5; 95% CI, 1.2–24.3) and males (*p* = 0.012; OR, 3.9; 95% CI, 1.3–14.2) (Fig. 4) and after stratifying by the different sequencing platforms; (*p* = 0.0001; OR, 4.2; 95% CI, 2.0–∞; by the CMH chi-squared test); in both the HiSeq 2500 (*p* = 0.003; OR, 5.0; 95% CI, 1.6–21.2) and the NovaSeq 6000 (*p* = 0.045; OR, 3.4; 95% CI, 1.0–14.3), (Fig. 4). To account for potential differences in temporal viral exposure among the older LTNP group (see “Methods“), we also evaluated the frequency of *Anelloviridae* among an external WGS dataset of 33 adults from Uganda of unknown HIV status. We did not find any evidence that these adults had a higher frequency of *Anelloviridae* than paediatric populations, with the frequency among adult controls (0.03) being similar to that observed in paediatric RPs (0.04) and comparable to, although slightly lower than, the combined paediatric LTNP group (0.12, n.s.), even after stratifying by country. (Supplementary Fig. 3).

## Discussion

Previous studies of HIV-infected individuals as they progress to AIDS have observed alterations in the virome, and more specifically, a reported increase in TTV viral loads^37,38^. However, there is limited information on how the host–virome of otherwise healthy HIV-infected children may influence the rate at which progression to AIDS occurs, given that viral co-infection has been consistently reported to influence the host’s immune state^18,39,40^. WES has and is being used to interrogate a variety of common and complex disease traits^41–43^; however, the full potential of the data generated has only rarely been leveraged to understand disease pathogenesis. Here, we demonstrate the added value of utilising off-target WES reads to investigate underlying differences in the blood viral-composition between individuals with varying rates of disease progression. Our observations of differences in WES-derived viral populations and *Anelloviridae* species between LTNP and RP groups provide an essential contribution to understanding the potential drivers of disease progression in HIV and a working framework for similar interrogations in other disease states.

We observed that the Uganda samples had higher viral diversity than the Botswana samples. However, this could be due to differences in the blood virome due to geography^26,44^. It may also suggest subtle upstream differences during sample preparation even though both study sites followed the same protocols and reagents for DNA collection.

We found LTNPs to have a significantly higher burden of viral reads than RPs, suggesting a possible role for the virome composition in AIDS progression. Commensal bacteria have been reported to prime the immune response in a way that offers cross-protection against pathogenic infections; similarly, viruses may delay HIV disease progression^18,39^. Our approach offers the potential for an agnostic interrogation that allows for the identification of significantly enriched species that might be specific to the population under study—much as genome-wide association studies have surpassed candidate gene studies. The growing utility of whole-genome datasets for complex trait studies suggests that exploring off-target reads for virome typing will yield even more comprehensive appraisals and become more commonplace over time.

In our dataset, *Anelloviridae* identification was strongly associated with LTNPs. *Anelloviridae* is a family of highly prevalent and genetically diverse viruses discovered relatively recently^45^. In humans, there are three reported genera: TTV, TTMDV, and TTMV^46^. There are up to seven reported phylogenetic clades, or phenogroups, of TTV^47–50^, of which the species identified in our dataset clustered with genogroup 1. *Anelloviridae* is ubiquitous, being found in >90% of (otherwise healthy) adults worldwide, with no known cases of human pathogenicity^46^; however, recent studies have found a strong association with immune suppression or exposure to new antigens^51^, and have propositioned this family of viruses as a marker for immune function^52–54^. In this regard, the statistical association of *Anelloviridae* with LTNP may appear somewhat paradoxical since an increased *Anelloviridae* viral load is associated with immune suppression^55^. This may be due to a subtle immune deficiency that is not present in RPs who have had longer ART. It may also in part be explained by recent findings that in children, reduced immune activation is associated with LTNP^16^, presumably via reduced expression of CCR5, a primary HIV-1 target. As such, we postulate that the higher *Anelloviridae* presence could be a marker of reduced immune activation in the paediatric HIV context.

Because of the time-dependent nature of disease progression, there are inherent confounders to this study; at the time of sample collection, the median age of LTNPs was, as expected, 7 years older than RPs (Table 1). This age difference makes it difficult to know whether our findings are primary (viral burden contributes to infection control) or secondary—the more prolonged environmental exposure of older LTNPs allowed for a more significant burden than the younger RPs. Although adults may have a higher TTV prevalence than children^56^, this association may depend on the study population. The TTV association with age primarily occurs within the first few years after birth, thereafter decreasing or stabilising into adulthood^57,58^; as such, in communities with a high prevalence of TTV, such as in developing countries, adults do not exhibit a higher TTV frequency since most individuals would have been infected during the first years of life^53,59^. Similarly, the frequency of *Anelloviridae* in our external adult Ugandan dataset suggests that temporal exposure may not be a major confounder of our result. However, at the time of sample collection, the median duration of HAART was significantly longer in the RPs than LTNPs (Table 1), which may influence host-viral diversity, and more specifically, *Anelloviridae* viral load. Although antivirals can affect the human virome structure^55^, antiretrovirals should, in theory, only affect RNA viruses. Although we cannot completely discount indirect methods by which prolonged HAART duration could affect *Anelloviridae* viral load, a recent study showed a lack of correlation between *Anelloviridae* viral load and increased T-cell counts or activated T cells in patients receiving ART^60^. There is also a theoretical possibility of LTNP misclassification due to a misdiagnosis of HIV-2 as HIV-1^61^, as HIV-2 is less virulent than HIV-1; we detected HIV-2 in 7 samples, 5 of which were LTNPs; however, none of the samples with *Anelloviridae* also had HIV-2 detected.

Our expectation is that our findings are most likely to reflect true viral infection, as opposed to potential environmental contaminants^26^; however, as we cannot completely rule out the latter potential, the influence of the blood virome on HIV-1 disease progression would benefit from a longitudinal prospective, rather than retrospective cohort study, in which the blood virome at baseline is known. In addition, the viral reads in this study were detected through sequence alignment, which limited our ability to detect viruses that may not be adequately represented by the reference sequences in the databases^62^. Such a study could therefore benefit from more recent viral capture panels such as ViroCap^63^ and VirCapSeq-VERT^64^ that increase viral reads by 100–10,000-fold and by using bioinformatics approaches that do not depend on sequence similarity for virus detection^65–67^. That said, such a longitudinal study is currently not feasible given changes to antiretroviral treatment guidelines whereby paediatric patients now receive ART at diagnosis, meaning LTNP cohorts similar to ours can no longer be clearly identified.

Given that TTV infections can occur as soon as 3 months postpartum^56–59^ and TTV is a T-lymphotropic virus^52,68^, we speculate that early TTV infection could play an active role in paediatric HIV disease progression by reducing the levels of immune activation and viral replication^15,16^. In vitro studies have demonstrated that TTV microRNA can interfere with interferon signalling^69^, a pathway important in inflammation and progression to AIDS during chronic HIV infection^70–72^. In addition, TTV has two main open reading frames (ORFs), ORF1 and ORF2, of which ORF2 suppresses the NF-κB pathways via interaction with IκB kinases^73^. Because the NF-κB signalling pathway is central to HIV-1 gene expression^74^, suppression of NF-κB activity could slow the rate of HIV-1 replication^75,76^. ORF2-mediated suppression of NF-κB activity could also lead to fewer activated CD4 T cells^77,78^, resulting in LTNP^16,79^, or may result in reduced pro-inflammatory cytokines such as TNFα slowing the disease progression^80^. Understanding the mechanistic underpinnings of this association between *Anelloviridae* and LTNP thus has the potential to catalyse research in this area further.

In conclusion, among extracted viral sequences from WES data, we identify an association between the viral species TTV and LTNP status. Our results are consistent with previous studies suggesting TTV as a biomarker for immune status and should stimulate consideration of TTV as a potential biomarker for HIV long-term non-progression.

## Methods

### Study participants

The study characteristics of the Collaborative African Genomics Network (CAfGEN) cohort have been previously described^81^, but, in brief, it includes children (aged 0–18 years) with laboratory-confirmed evidence of HIV-1 infection who were registered for care at the Baylor College of Medicine Children’s Foundation in Kampala, Uganda, or the Botswana-Baylor Children’s Clinical Centre of Excellence in Gaborone, Botswana, both of which are the major centres for paediatric HIV care in their respective countries. Potential participants meeting clinical criteria were retrospectively identified from electronic health records dating back more than 20 years in the two centres of excellence. After obtaining informed consent and assent, participants were enrolled as part of a retrospective case–control study investigating the genetics of paediatric HIV disease progression as part of CAfGEN^81–83^—a collaborative centre of the Human Heredity and Health in Africa (H3Africa) Consortium^84^. Electronic health records in both centres were retrospectively queried to identify individuals meeting World Health Organization (WHO) clinical and immunologic criteria for RPs, i.e. those with (1) two or more CD4 T-cell proportion values < 15% within 3 years after birth, with no value > 15% afterwards in the absence of ART; (2) ART initiated within 3 years after birth, and at least one preceding CD4 < 15%; and (3) AIDS-defining illness (CDC Cat 3 or WHO Stage 3/4) and LTNPs—children asymptomatic > 10 years after initial infection (birth) who had not met the criteria for ART initiation.

### Whole-exome sequencing

Peripheral blood was collected from LTNPs (*n* = 391) or RPs (*n* = 422) and gDNA extracted using the PAXgene Blood DNA kit (Qiagen, USA). Exome reads were captured and enriched from gDNA and subsequently sequenced at the Human Genome Sequencing Center, Baylor College of Medicine, as previously described^85–87^. Briefly, gDNA samples were processed and quantified to meet quality control criteria and were then fragmented prior to exon enrichment using NimbleGen VCRome 2.1 (rebalanced probe) capture reagent and subsequent ligation of indexed adaptors to allow for multiplexed sequencing. The libraries were sequenced in seven batches using a paired-end, 100 base-pair read length protocol on a HiSeq 2500 (round 1, batches 1–3) and subsequently, a NovaSeq 6000 Illumina platform (round 2, batches 4–7) (Illumina, San Diego, CA) using the TruSeq SBS Kits (Illumina, San Diego, CA) per manufacturer’s instructions, and base-calling files were converted to FASTQ files using bclToFastq (version 1.8.3). There were no differences in the distribution of sex, country of origin, and phenotype across the sequencing platforms (Supplementary Data 1).

### Virus detection

VirusFinder 2 workflow^88^ was used to extract viral reads from WES data with additional parameters summarised in Supplementary Table 1. Raw sequencing reads (FASTQ files) were aligned to the human reference genome (UCSC hg19) using Bowtie2^89^. All unmapped reads were then used for virus detection by BLAST-searching against a virus dataset^90^ that contains viruses of all known classes (*n* = 32,102). Viral reads were assembled de novo into contigs, and non-human viral contigs were mapped to the virus dataset and ranked based on the alignment scores.

We standardised viral nomenclature using the International Committee on Taxonomy of Viruses Master Species Lists (https://talk.ictvonline.org/files/master-species-lists/). Because phiX DNA (from *Enterobacteria phage phiX174* (*Microviridae*)) was spiked-in as a sequencing control, we excluded *Microviridae* from downstream analyses; however, we used the equal distribution of *Microviridae* reads between LTNPs and RPs and between Uganda and Botswana samples (Supplementary Data 1) as a quality check for potential sequencing biases between cases and controls, or between the different countries of origin.

### Validation and phylogenetic analysis of *Anelloviridae*

To validate the *Anelloviridae* reads, we carried out PCR amplification using the following primers as previously described; TTxsense: 5′-CACTTCCGAATGGYWGAGTTT-3′ and TTxrev: 5′-TCCCGAGCCCGAATTGCCCCT-3′^91^, modified to use 1 μM of both forward and reverse primers, and HotStar HiFidelity Polymerase (Qiagen, USA) according to the manufacturer’s recommendations. The expected PCR product was 110–120 bp, depending on the viral strain amplified. The PCR product was purified using the QIAquick Gel Extraction Kit (Qiagen, USA) according to the manufacturer’s recommendations and sequenced using Dideoxy (Sanger) sequencing, followed by a BLAST search to confirm that the sequences were from TTV.

To determine the phylogenetic relationship of TTV, we performed a CLUSTALW multiple sequence alignment (Unipro UGENE v34.0 software) of the study samples with other known TTV genogroups consisting of 17 GenBank sequences representing TTV genogroups 1–5^48–50^ (Supplementary Data 1). We trimmed ambiguous alignments on 5′-end and 3′-end and inferred the phylogenetic relationship using the maximum likelihood method and Tamura–Nei model in MEGA v10.1.8.

### External control dataset

Because LTNPs were older than the RPs, and thus, would have had a longer temporal exposure to environmental viruses (see “Results”), we utilised an external dataset of WGS from 33 adults from central Uganda, collected under the TrypanoGEN study^92,93^, and similarly sequenced on the Illumina HiSeq 2500 (Illumina, San Diego, CA) as age-related controls. Unmapped reads from this dataset were also run through the VirusFinder v2.0 pipeline with identical parameters to the WES dataset. Tae et al.^27^ looked at individuals who had been sequenced using both WES and WGS and found the ratios of unmapped to mapped reads to be the same between WGS and WES.

### Ethics declarations

Institutional Review Board (IRB) approval for the CAfGEN project was obtained from the School of Biomedical Sciences Higher Degrees Research and Ethics Committee (SBS-REC) (Ref no: SBS 112), Uganda National Council for Science and Technology (Ref no: HS 1566), Health Research and Development Committee, Ministry of Health and Wellness, Botswana (Ref no: HPDME 13/18/1 IX (484), and the IRB for Human Subject Research for Baylor College of Medicine and Affiliated Hospitals (BCM IRB) (Ref no: H-32788). We obtained written informed consent from all adult study participants and the primary caregivers of children. In addition, children from appropriate age groups gave assent to participate in the study.

### Statistical analyses

We compared the means or medians for continuous variables using the Welch’s two-sample *t*-test or Wilcoxon rank-sum test, respectively, and a two-tailed Fisher’s exact test with ORs and 95% CI for categorical variables. We used the false-discovery rate (FDR) method^94^ to correct for multiple comparisons and, where appropriate, a two-tailed CMH chi-squared test to calculate the common ORs to control for confounding. Where applicable, we estimated the OR and 95% CI from the GLMs by exponentiating the coefficients. The *t*-test, Fisher’s exact test, CMH, and GLM were computed in R (version 3.6.3). The Hutcheson *t*-test^36^ was used to compare the Shannon diversity index of the viral communities between groups^95^. The statistical significance level was set at 0.05.

### Reporting summary

Further information on research design is available in the Nature Research Reporting Summary linked to this article.

## Supplementary information

Supplementary Information

Supplementary Data 1

Reporting Summary

## Data Availability

The virus list that was generated for analysis is available in Supplementary Data 1. The WES datasets generated and analysed during the current study will be deposited into the European Genome-phenome Archive (https://www.ebi.ac.uk/ega/) [phase 1 samples are currently in EGA (accession number: EGAS00001002656) and available for download https://ega-archive.org/studies/EGAS00001002656], consistent with the H3Africa Consortium consensus agreement. Data will be made available through the H3Africa Data and Biospecimen Access Committee upon reasonable request from validated researchers (https://www.h3abionet.org/resources/h3africa-archive).

## Code availability

Scripts used for data analysis are available at https://github.com/savannahmwesigwa/CAFGEN.

## References

[1] Joint United Nations Programme on HIV/AIDS (UNAIDS). *Regional Statistics—2015* (Joint United Nations Programme on HIV/AIDS (UNAIDS), 2016). http://www.unaids.org/sites/default/files/media_asset/UNAIDS_FactSheet_en.pdf. 12349391

[2] Joint United Nations Programme on HIV/AIDS (UNAIDS). *Fact Sheet December 2019: Global HIV Statistics—World AIDS Day 2019* (Joint United Nations Programme on HIV/AIDS (UNAIDS), 2019). https://www.unaids.org/sites/default/files/media_asset/UNAIDS_FactSheet_en.pdf.

[3] Warszawski J (2007). Long-term nonprogression of HIV infection in children: evaluation of the ANRS Prospective French Pediatric Cohort. Clin. Infect. Dis..

[4] Dean M (1996). Genetic restriction of HIV-1 infection and progression to AIDS by a deletion allele of the CKR5 structural gene. Science.

[5] Hartley O (2004). Medicinal chemistry applied to a synthetic protein: development of highly potent HIV entry inhibitors. Proc. Natl Acad. Sci. USA.

[6] Jiang Y (2013). KIR3DS1/L1 and HLA-Bw4-80I are associated with HIV disease progression among HIV typical progressors and long-term nonprogressors. BMC Infect. Dis..

[7] Martin MP (2002). Epistatic interaction between KIR3DS1 and HLA-B delays the progression to AIDS. Nat. Genet..

[8] Wayengera M (2015). Genomic-regulation of active retroviral elements as a model for HIV cure. J. Antivir. Antiretrovir..

[9] Casado C (2010). Host and viral genetic correlates of clinical definitions of HIV-1 disease progression. PLoS ONE.

[10] Berhane R (1997). Growth failure as a prognostic indicator of mortality in pediatric HIV infection. Pediatrics.

[11] Koethe JR, Heimburger DC (2010). Nutritional aspects of HIV-associated wasting in sub-Saharan Africa. Am. J. Clin. Nutr..

[12] Baum MK (2013). Effect of micronutrient supplementation on disease progression in asymptomatic, antiretroviral-naive, HIV-infected adults in Botswana: a randomized clinical trial. JAMA.

[13] Wood A, Armour D (2005). The discovery of the CCR5 receptor antagonist, UK-427,857, a new agent for the treatment of HIV infection and AIDS. Prog. Med. Chem..

[14] Gurdasani D (2014). The African Genome Variation Project shapes medical genetics in Africa. Nature.

[15] Ssewanyana I (2007). Profile of T cell immune responses in HIV‐infected children from Uganda. J. Infect. Dis..

[16] Muenchhoff M (2016). Nonprogressing HIV-infected children share fundamental immunological features of nonpathogenic SIV infection. Sci. Transl. Med..

[17] Rouse BT, Sehrawat S (2010). Immunity and immunopathology to viruses: what decides the outcome?. Nat. Rev. Immunol..

[18] Cadwell K (2015). The virome in host health and disease. Immunity.

[19] Münz C, Lünemann JD, Getts MT, Miller SD (2009). Antiviral immune responses: triggers of or triggered by autoimmunity?. Nat. Rev. Immunol..

[20] Draborg AH, Duus K, Houen G (2013). Epstein-Barr virus in systemic autoimmune diseases. Clin. Dev. Immunol..

[21] Gordon A (2019). Prior dengue virus infection and risk of Zika: a pediatric cohort in Nicaragua. PLoS Med..

[22] Turci M (2006). Coinfection with HIV-1 and human T-Cell lymphotropic virus type II in intravenous drug users is associated with delayed progression to AIDS. J. Acquir. Immune Defic. Syndr..

[23] Beilke MA (2004). Clinical outcomes and disease progression among patients coinfected with HIV and human T lymphotropic virus types 1 and 2. Clin. Infect. Dis..

[24] Tang K-W, Larsson E (2017). Tumour virology in the era of high-throughput genomics. Philos. Trans. R. Soc. Lond. B. Biol. Sci..

[25] Samuels DC (2013). Finding the lost treasures in exome sequencing data. Trends Genet..

[26] Moustafa A (2017). The blood DNA virome in 8,000 humans. PLoS Pathog..

[27] Tae H, Karunasena E, Bavarva JH, McIver LJ, Garner HR (2014). Large scale comparison of non-human sequences in human sequencing data. Genomics.

[28] Strong MJ (2016). A comprehensive next generation sequencing-based virome assessment in brain tissue suggests no major virus—tumor association. Acta Neuropathol. Commun..

[29] Sulonen AM (2011). Comparison of solution-based exome capture methods for next generation sequencing. Genome Biol..

[30] Guo Y (2012). Exome sequencing generates high quality data in non-target regions. BMC Genom..

[31] Asan (2011). Comprehensive comparison of three commercial human whole-exome capture platforms. Genome Biol..

[32] Martinez-Hernandez F (2017). Single-virus genomics reveals hidden cosmopolitan and abundant viruses. Nat. Commun..

[33] Desfarges, S. & Ciuffi, A. Viral integration and consequences on host gene expression. in *Viruses: Essential Agents of Life*, Vol. 9789400748996, 147–175 (Springer Netherlands, 2012).

[34] Palmisano L, Vella S (2011). A brief history of antiretroviral therapy of HIV infection: success and challenges. Ann. Ist. Super. Sanita.

[35] Demeulemeester J (2014). HIV-1 integrase variants retarget viral integration and are associated with disease progression in a chronic infection cohort. Cell Host Microbe.

[36] Hutcheson K (1970). A test for comparing diversities based on the Shannon formula. J. Theor. Biol..

[37] Monaco CL (2016). Altered virome and bacterial microbiome in human immunodeficiency virus-associated acquired immunodeficiency syndrome. Cell Host Microbe.

[38] Thom K, Petrik J (2007). Progression towards AIDS leads to increased torque teno virus and torque teno minivirus titers in tissues of HIV infected individuals. J. Med. Virol..

[39] Duerkop BA, Hooper LV (2013). Resident viruses and their interactions with the immune system. Nat. Immunol..

[40] Virgin HW, Wherry EJ, Ahmed R (2009). Redefining chronic viral infection. Cell.

[41] Flannick J (2019). Exome sequencing of 20,791 cases of type 2 diabetes and 24,440 controls. Nature.

[42] Bis, J. C. et al. Whole exome sequencing study identifies novel rare and common Alzheimer’s-associated variants involved in immune response and transcriptional regulation. *Mol. Psychiatry* 1–17 (2018). 10.1038/s41380-018-0112-7. 10.1038/s41380-018-0112-7 PMC6375806 30108311

[43] Sabo A (2017). Exome sequencing reveals novel genetic loci influencing obesity-related traits in Hispanic children. Obesity.

[44] Zuo T (2020). Human-gut-DNA virome variations across geography, ethnicity, and urbanization. Cell Host Microbe.

[45] Nishizawa T (1997). A novel DNA virus (TTV) associated with elevated transaminase levels in posttransfusion hepatitis of unknown etiology. Biochem. Biophys. Res. Commun..

[46] Nishiyama S (2014). Identification of novel anelloviruses with broad diversity in UK rodents. J. Gen. Virol..

[47] Sarairah H, Bdour S, Gharaibeh W (2020). The molecular epidemiology and phylogeny of torque teno virus (TTV) in Jordan. Viruses.

[48] Hsiao K-L, Wang L-Y, Lin C-L, Liu H-F (2016). New phylogenetic groups of torque teno virus identified in Eastern Taiwan Indigenes. PLoS ONE.

[49] Biagini P (2009). Classification of TTV and related viruses (anelloviruses). Curr. Top. Microbiol. Immunol..

[50] Peng YH (2002). Analysis of the entire genomes of thirteen TT virus variants classifiable into the fourth and fifth genetic groups, isolated from viremic infants. Arch. Virol..

[51] Maggi F (2005). Blood levels of TT virus following immune stimulation with influenza or hepatitis B vaccine. J. Med. Virol..

[52] Focosi D, Macera L, Boggi U, Nelli LC, Maggi F (2015). Short-term kinetics of torque teno virus viraemia after induction immunosuppression confirm T lymphocytes as the main replication-competent cells. J. Gen. Virol..

[53] Tyschik EA, Rasskazova AS, Degtyareva AV, Rebrikov DV, Sukhikh GT (2018). Torque teno virus dynamics during the first year of life. Virol. J..

[54] Jaksch P (2018). Torque teno virus as a novel biomarker targeting the efficacy of immunosuppression after lung transplantation. J. Infect. Dis..

[55] De Vlaminck I (2013). Temporal response of the human virome to immunosuppression and antiviral therapy. Cell.

[56] Saback FL (1999). Age-specific prevalence and transmission of TT virus. J. Med. Virol..

[57] Komatsu H (2004). TTV infection in children born to mothers infected with TTV but not with HBV, HCV, or HIV. J. Med. Virol..

[58] Hsu HY, Ni YH, Chen HL, Kao JH, Chang MH (2003). TT virus infection in healthy children, children after blood transfusion, and children with non-A to E hepatitis or other liver diseases in Taiwan. J. Med. Virol..

[59] Davidson F (1999). Early acquisition of TT Virus (TTV) in an area endemic for TTV infection. J. Infect. Dis..

[60] Li L (2015). Virome analysis of antiretroviral-treated HIV patients shows no correlation between T-cell activation and anelloviruses levels. J. Clin. Virol..

[61] Migueles SA, Connors M (2010). Long-term nonprogressive disease among individuals with untreated HIV infection—reply. JAMA.

[62] Krishnamurthy SR, Wang D (2017). Origins and challenges of viral dark matter. Virus Res..

[63] Wylie TN (2015). Enhanced virome sequencing using targeted sequence capture enhanced virome sequencing using sequence capture. Genome Res..

[64] Briese T (2015). Virome capture sequencing enables sensitive viral diagnosis and comprehensive virome analysis. MBio.

[65] Ren J, Ahlgren NA, Lu YY, Fuhrman JA, Sun F (2017). VirFinder: a novel k-mer based tool for identifying viral sequences from assembled metagenomic data. Microbiome.

[66] Seguritan V (2012). Artificial neural networks trained to detect viral and phage structural proteins. PLoS Comput. Biol..

[67] Skewes-Cox P, Sharpton TJ, Pollard KS, DeRisi JL (2014). Profile hidden Markov models for the detection of viruses within metagenomic sequence data. PLoS ONE.

[68] Fabrizio M (2008). Changes in CD8^+^57+ T lymphocyte expansions after autologous hematopoietic stem cell transplantation correlate with changes in torquetenovirus viremia. Transplantation.

[69] Kincaid RP, Burke JM, Cox JC, de Villiers EM, Sullivan CS (2013). A human torque teno virus encodes a microRNA that inhibits interferon signaling. PLoS Pathog..

[70] Doyle T, Goujon C, Malim MH (2015). HIV-1 and interferons: who’s interfering with whom?. Nat. Rev. Microbiol..

[71] Utay NS, Douek DC (2016). Interferons and HIV infection: the good, the bad, and the ugly. Pathog. Immun..

[72] Nganou-Makamdop K, Douek DC (2019). Manipulating the interferon signaling pathway: implications for HIV infection. Virol. Sin..

[73] Zheng H (2007). Torque teno virus (SANBAN isolate) ORF2 protein suppresses NF-κB pathways via interaction with IκB kinases. J. Virol..

[74] Hiscott J, Kwon H, Génin P (2001). Hostile takeovers: viral appropriation of the NF-κB pathway. J. Clin. Investig..

[75] Miyake A (2010). Inhibition of active HIV-1 replication by NF-κB inhibitor DHMEQ. Microbes Infect..

[76] Shi T, Wilhelm E, Bell B, Dumais N (2017). Nf-κb-dependent inhibition of HIV-1 transcription by withaferin A. HIV Curr. Res..

[77] Liu T, Zhang L, Joo D, Sun SC (2017). NF-κB signaling in inflammation. Signal Transduct. Target. Therapy.

[78] Oh H, Ghosh S (2013). NF-κB: roles and regulation in different CD4^+^ T-cell subsets. Immunol. Rev..

[79] Deeks SG, Walker BD (2004). The immune response to AIDS virus infection: good, bad, or both?. J. Clin. Investig..

[80] Vaidya SA (2014). Tumor necrosis factor α is associated with viral control and early disease progression in patients with HIV type 1 infection. J. Infect. Dis..

[81] Mboowa G (2018). The Collaborative African Genomics Network (CAfGEN): applying genomic technologies to probe host factors important to the progression of HIV and HIV-tuberculosis infection in sub-Saharan Africa. AAS Open Res..

[82] Retshabile G (2018). Whole-exome sequencing reveals uncaptured variation and distinct ancestry in the Southern African population of Botswana. Am. J. Hum. Genet..

[83] Mlotshwa BC (2017). The Collaborative African Genomics Network training program: a trainee perspective on training the next generation of African scientists. Genet. Med..

[84] The H3Africa Consortium. (2014). Enabling the genomic revolution in Africa. Science.

[85] Lupski JR (2013). Exome sequencing resolves apparent incidental findings and reveals further complexity of SH3TC2 variant alleles causing Charcot-Marie-Tooth neuropathy. Genome Med..

[86] Yang Y (2013). Clinical whole-exome sequencing for the diagnosis of Mendelian disorders. N. Engl. J. Med..

[87] McMichael G (2015). Whole-exome sequencing points to considerable genetic heterogeneity of cerebral palsy. Mol. Psychiatry.

[88] Wang Q, Jia P, Zhao Z (2013). VirusFinder: software for efficient and accurate detection of viruses and their integration sites in host genomes through next generation sequencing data. PLoS ONE.

[89] Langmead B, Salzberg SL (2012). Fast gapped-read alignment with Bowtie 2. Nat. Methods.

[90] Bhaduri A, Qu K, Lee CS, Ungewickell A, Khavari PA (2012). Rapid identification of non-human sequences in high-throughput sequencing datasets. Bioinformatics.

[91] Bédarida S, Dutour O, Buzhilova AP, de Micco P, Biagini P (2011). Identification of viral DNA (*Anelloviridae*) in a 200-year-old dental pulp sample (Napoleon’s Great Army, Kaliningrad, 1812). Infect. Genet. Evol..

[92] Ilboudo H (2017). Introducing the TrypanoGEN biobank: a valuable resource for the elimination of human African trypanosomiasis. PLoS Negl. Trop. Dis..

[93] Mulindwa J (2020). High levels of genetic diversity within Nilo-Saharan populations: implications for human adaptation. Am. J. Hum. Genet..

[94] Benjamini Y, Hochberg Y (1995). Controlling the false discovery rate: a practical and powerful approach to multiple testing. J. R. Stat. Soc..

[95] Gardener, M. *Statistics for Ecologists Using R and Excel: Data Collection, Exploration, Analysis and Presentation (Data in the Wild)* (Pelagic Publishing, 2017).

