# Supplementary material for: Unmapped exome reads implicate a role for Anelloviridae in childhood HIV-1 long-term non-progression
Source: NPJ Genom Med. 2021 Mar 19;6:24. doi: 10.1038/s41525-021-00185-w (PMC7979878; doi:10.1038/s41525-021-00185-w)
Supplement: Supplementary file 1 — Supplementary Information [file 41525_2021_185_MOESM1_ESM.pdf]

Unmapped exome reads implicate a role for *Anelloviridae* in childhood HIV-1 long-term non-progression

Savannah Mwesigwa, Lesedi Williams, Gaone Retshabile, Eric Katagirya, Gerald Mboowa, Busisiwe Mlotshwa, Samuel Kyobe, David P. Kateete, Eddie Wampande, Misaki Wayengera, Sununguko Wata Mpoloka, Angella N. Mirembe, Ishmael Kasvosve, Koketso Morapedi, Grace P. Kisitu, Adeodata R. Kekitiinwa, Gabriel Anabwani, Moses L. Joloba, Enock Matovu, Julius Mulindwa, Harry Noyes, Gerrit Botha, Chester W. Brown, Graeme Mardon, Mogomotsi Matshaba, Neil Hanchard

Supplementary information

Supplementary Table 1 Summary of VirusFinder parameters used.

**Parameter**	**Setting**
Detection mode	sensitive
Flank region size	4000
Sensitivity level	3
Minimum contig length	100
BLASTN E-value threshold	0.05
Similarity threshold	0.8
Chop read length	25
Minimum Identity	80

Supplementary Table 2 Summary statistics comparing the diversity of viral populations. The Hutcheson t-test was used to compare the Shannon diversity index. The LTNPs showed higher diversity than RPs (**a**), and Uganda had higher diversity than Botswana (**b**).

**a**	**LTNP**	**RP**
Total	378	325
Richness	31	32
H	2.17392	1.837406
S^2H	0.006718	0.009139
t	2.672385	
Degrees of freedom	668	
critical value	1.963522	
p-value	0.007715	
95% CI	0.163926	0.191191

**b**	**Uganda**	**Botswana**
Total	399	307
Richness	37	29
H	2.396445	1.478662
S^2_H	0.006028	0.009386
t	7.392435	
Degrees of freedom	628	
critical value	1.963749	
p-value	4.62E-13	
95% CI	0.155275577	0.193762787

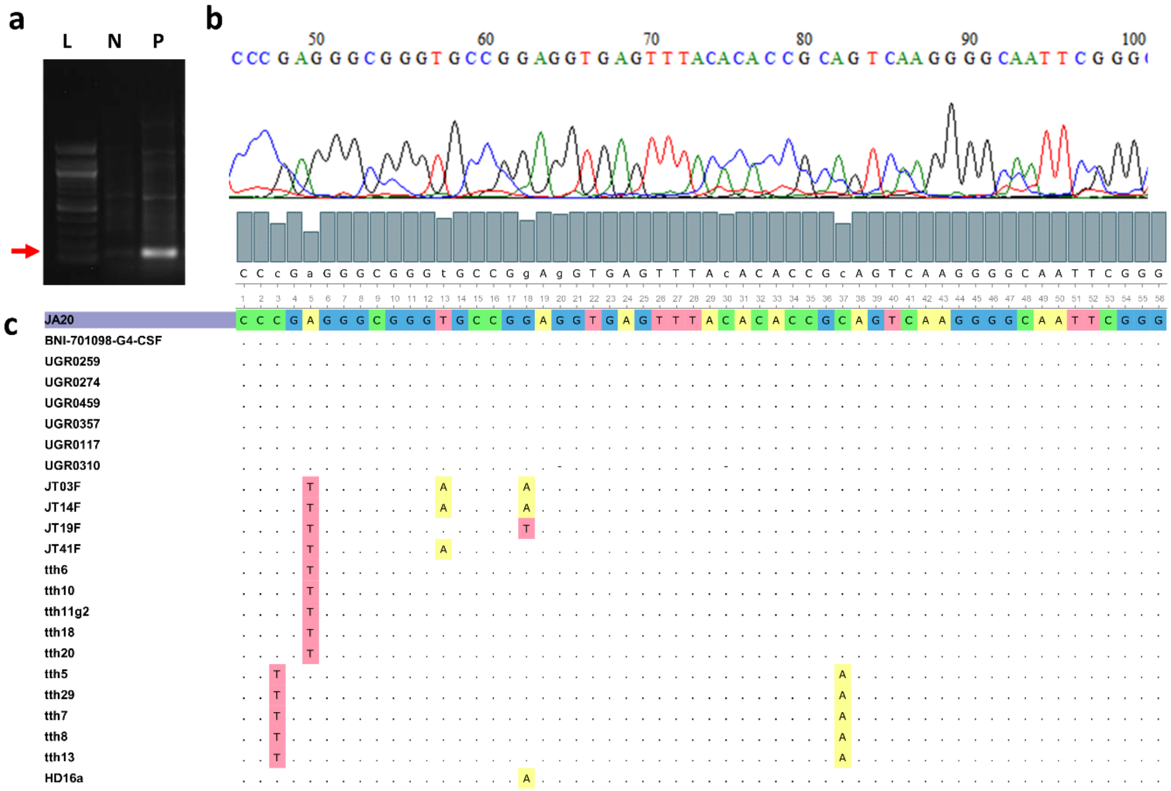

Supplementary Figure 1. Samples with TTV detected were validated with PCR followed by Sanger sequencing. a Representative gel of PCR amplification of a ~100bp TTV region (red arrow) carried out on both samples that had TTV detected (lane P) and random samples selected that did not have TTV detected in the WES dataset (lane N). **b** The TTV amplicon was Sanger sequenced to confirm presence of TTV. **c** The sequences were aligned with representative sequences for TTV genogroups 1-5, with JA20 (genogroup 1) set as the reference; dots represent consensus with the reference sequence. The Uganda samples show 100 percent identity to BNI-701098-G4-CSF and JA20 (shaded grey), which both belong to genogroup 1 TTV.

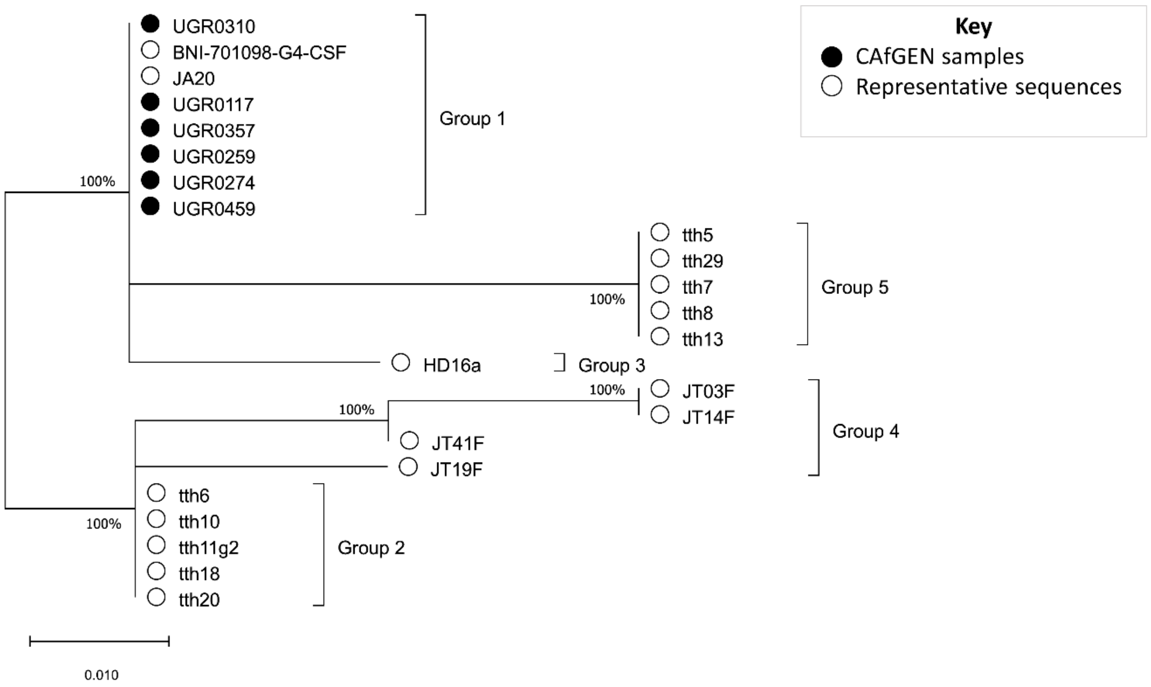

Supplementary Figure 2. The phylogeny of the TTV identified in the Ugandan samples. Phylogenetic analysis was inferred using the Maximum Likelihood method and the Tamura-Nei model. The TTV from the Uganda samples clustered with genogroup 1 TTV, BNI-701098-G4-CSF, and JA20.

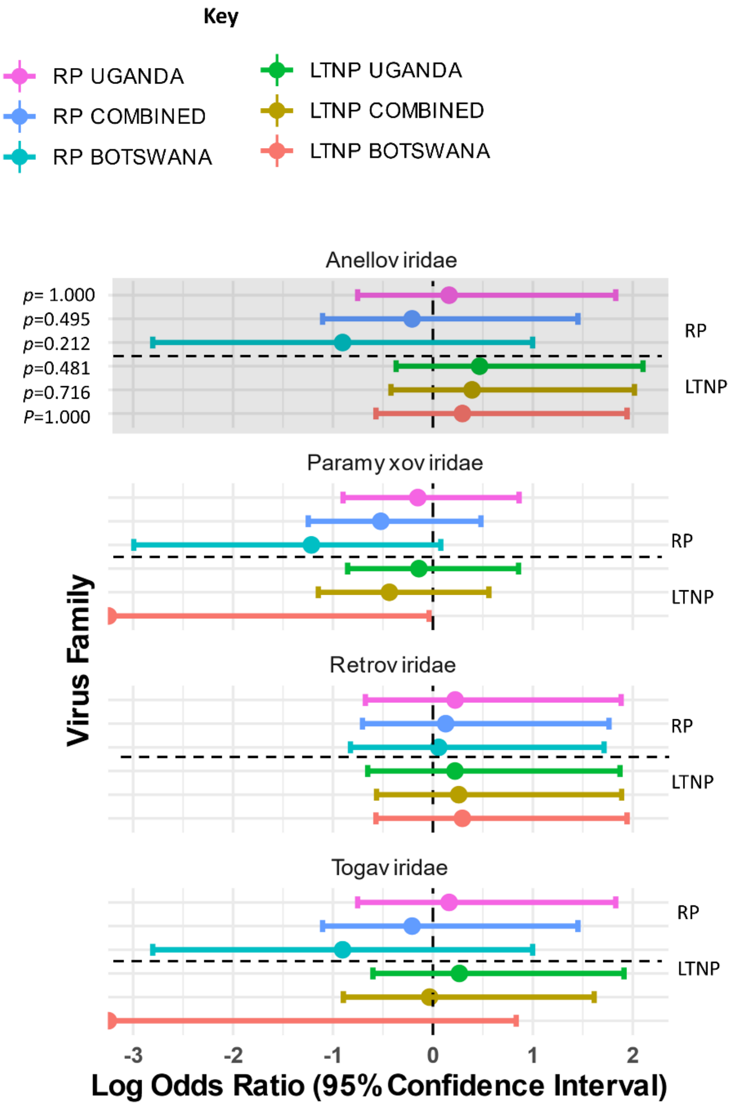

Supplementary Figure 3. Forest plot of comparing effect size of association of the virus families with pediatrics versus adults (as controls). To assess whether the increased association of Anelloviridae in the older LTNP group was due to their age, we compared the pediatric *CAfGEN* groups (stratified by country and disease progression) with an adult TrypanoGEN group. The Anelloviridae did not show any significant association with the adult group (shaded) but trended towards an association with LTNP (not statistically significant). P-values, odds ratios (95%CI) were calculated using Fisher’s exact test.

Supplementary Data 1. Raw output data from bioinformatics and statistical analyses
