# Supplementary material for: Unmapped exome reads implicate a role for Anelloviridae in childhood HIV-1 long-term non-progression
Source: NPJ Genom Med. 2021 Mar 19;6:24. doi: 10.1038/s41525-021-00185-w (PMC7979878; doi:10.1038/s41525-021-00185-w)
Supplement: Supplementary file 3 — Reporting Summary [file 41525_2021_185_MOESM3_ESM.pdf]

nature research

Corresponding author(s): Neil Hanchard
Last updated by author(s): Dec 2, 2020

Reporting Summary

Nature Research wishes to improve the reproducibility of the work that we publish. This form provides structure for consistency and transparency in reporting. For further information on Nature Research policies, see our Editorial Policies and the Editorial Policy Checklist.

Statistics

For all statistical analyses, confirm that the following items are present in the figure legend, table legend, main text, or Methods section.

n/a	Confirmed	
☐	☒	The exact sample size (*n*) for each experimental group/condition, given as a discrete number and unit of measurement
☒	☐	A statement on whether measurements were taken from distinct samples or whether the same sample was measured repeatedly
☐	☒	The statistical test(s) used AND whether they are one- or two-sided<br>*Only common tests should be described solely by name; describe more complex techniques in the Methods section.*
☐	☒	A description of all covariates tested
☐	☒	A description of any assumptions or corrections, such as tests of normality and adjustment for multiple comparisons
☐	☒	A full description of the statistical parameters including central tendency (e.g. means) or other basic estimates (e.g. regression coefficient) AND variation (e.g. standard deviation) or associated estimates of uncertainty (e.g. confidence intervals)
☐	☒	For null hypothesis testing, the test statistic (e.g. *F*, *t*, *r*) with confidence intervals, effect sizes, degrees of freedom and *P* value noted<br>*Give P values as exact values whenever suitable.*
☒	☐	For Bayesian analysis, information on the choice of priors and Markov chain Monte Carlo settings
☒	☐	For hierarchical and complex designs, identification of the appropriate level for tests and full reporting of outcomes
☐	☒	Estimates of effect sizes (e.g. Cohen's *d*, Pearson's *r*), indicating how they were calculated

Our web collection on statistics for biologists contains articles on many of the points above.

Software and code

Policy information about availability of computer code

Data collection: MySQL version 5

Data analysis: bclToFastq (version 1.8.3), bwa (version 0.7), R (version 3.6.3), VirusFinder 2, Excel
Scripts used for data analysis are available at https://github.com/savannahmwesigwa/CAFGEN

For manuscripts utilizing custom algorithms or software that are central to the research but not yet described in published literature, software must be made available to editors and reviewers. We strongly encourage code deposition in a community repository (e.g. GitHub). See the Nature Research guidelines for submitting code & software for further information.

Data

Policy information about availability of data

All manuscripts must include a data availability statement. This statement should provide the following information, where applicable:
- Accession codes, unique identifiers, or web links for publicly available datasets
- A list of figures that have associated raw data
- A description of any restrictions on data availability

The datasets generated and analyzed during the current study will be deposited into the European Genome-phenome Archive (https://www.ebi.ac.uk/ega/) [phase 1 samples are currently in EGA (accession number: EGAS00001002656) and available for download https://ega-archive.org/studies/EGAS00001002656], consistent with the H3Africa Consortium consensus agreement. Data will be made available through the H3Africa Data and Biospecimen Access Committee (DBAC) upon reasonable request from validated researchers (https://www.h3abionet.org/resources/h3africa-archive).

Field-specific reporting

Please select the one below that is the best fit for your research. If you are not sure, read the appropriate sections before making your selection.

☒ Life sciences ☐ Behavioural & social sciences ☐ Ecological, evolutionary & environmental sciences

For a reference copy of the document with all sections, see nature.com/documents/nr-reporting-summary-flat.pdf

Life sciences study design

All studies must disclose on these points even when the disclosure is negative.

Sample size	Assuming alpha=0.05, the sample size had a 97.5% power to detect a relative risk of 4.
Data exclusions	We excluded viral families that were found in less than 1 percent of the samples to rule out possible contamination. We excluded Microviridae from downstream analyses because phiX DNA (from Enterobacteria phage phiX174 (Microviridae)) is used as spiked-in as a sequencing control.
Replication	One batch of samples was sequenced on the Illumina HiSeq 2500 and a second batch was sequenced on the NovaSeq 6000 Illumina platform, both showed an association of Anelloviridae with LTNP status. Additionally samples came for two countries, Uganda and Botswana which also showed the same trend though only statistically significant in the Botswana group.
Randomization	Participants were enrolled on a rolling basis until target sample sizes were met. We used a a logistic regression analysis using a generalized linear model (GLM) with a binomial distribution, and controlled for age at sample collection, sex, country of origin, and sequencing platform as covariates.
Blinding	Blinding was not possible as this was a retrospective study, however during the sample preparation and sequencing process, all investigators and technologists were blinded from the sample phenotypes.

Reporting for specific materials, systems and methods

We require information from authors about some types of materials, experimental systems and methods used in many studies. Here, indicate whether each material, system or method listed is relevant to your study. If you are not sure if a list item applies to your research, read the appropriate section before selecting a response.

Materials & experimental systems

n/a	Involved in the study	
☒	☐	Antibodies
☒	☐	Eukaryotic cell lines
☒	☐	Palaeontology and archaeology
☒	☐	Animals and other organisms
☐	☒	Human research participants
☒	☐	Clinical data
☒	☐	Dual use research of concern

Methods

n/a	Involved in the study	
☒	☐	ChIP-seq
☒	☐	Flow cytometry
☒	☐	MRI-based neuroimaging

Human research participants

Policy information about studies involving human research participants

Population characteristics	Participants were aged 0-18 years (median age 13 years) with laboratory-confirmed evidence of HIV-1 infection from Uganda (45%) and Botswana (55%).<br>48% were classified as LTNPs and had a median 156 month to disease progression and had been on antiretroviral therapy (ART) for a median 19 months while 52% were classified as RPs with a median 17 months to disease progression and had been on ART for a median 98 months.<br>52% of the participants were female and 48% were male.
Recruitment	Electronic health records were retrospectively queried to identify individuals meeting World Health Organization (WHO) clinical and immunologic criteria for RPs, i.e., those with (a) two or more CD4 T cell proportion values <15% within three years after birth, with no value >15% afterward in the absence of ART; (b) ART initiated within three years after birth, and at least one preceding CD4<15%; (c) AIDS-defining illness (CDC Cat 3 or WHO Stage 3/4); and LTNPs were children asymptomatic >10 years after initial infection (birth) who had not met the criteria for ART initiation.
Ethics oversight	Institutional Review Board (IRB) approval for the CAfGEN project was obtained from The School of Biomedical Sciences Higher Degrees Research and Ethics Committee (SBS-REC) (Ref no: SBS 112), Uganda National Council for Science and Technology (UNCST) (Ref no: HS 1566), Health Research and Development Committee (HRDC), Ministry of Health and

Wellness, Botswana (Ref no: HPDME 13/18/1 IX (484), and the Institutional Review Board for Human Subject Research for Baylor College of Medicine and Affiliated Hospitals (BCM IRB) (Ref no: H-32788).

Note that full information on the approval of the study protocol must also be provided in the manuscript.
